# Evaluation of several ionic liquids for in situ hydrolysis of empty fruit bunches by locally-produced cellulase

**DOI:** 10.1007/s13205-016-0440-8

**Published:** 2016-06-08

**Authors:** Amal Ahmed Elgharbawy, Md. Zahangir Alam, Nassereldeen Ahmad Kabbashi, Muhammad Moniruzzaman, Parveen Jamal

**Affiliations:** 1 Department of Biotechnology Engineering, Faculty of Engineering, Bioenvironmental Engineering Research Centre (BERC), International Islamic University Malaysia, 50728 Kuala Lumpur, Malaysia; 2 Centre of Research in Ionic Liquids (CORIL), Chemical Engineering Department, Universiti Teknologi Petronas, 32610 Bandar Seri Iskandar, Malaysia

**Keywords:** Activity, Cellulase, Compatibility, Ionic liquid, Lignocellulose, Pretreatment

## Abstract

Lignocellulosic biomasses, exhibit resistance to enzymatic hydrolysis due to the presence of lignin and hemicellulose. Ionic liquids proved their applicability in lignin degradation, however, ionic liquid removal has to be performed to proceed to hydrolysis. Therefore, this study reports an in situ hydrolysis of empty fruit bunches (EFB) that combined an ionic liquid (IL) pretreatment and enzymatic hydrolysis. For enzyme production, palm kernel cake (PKC) was used as the primary media for microbial cellulase (PKC-Cel) from *Trichoderma reesei* (RUTC30). The obtained enzyme exhibited a promising stability in several ionic liquids. Among few, in choline acetate [Cho]OAc, PKC-Cel retained 63.16 % of the initial activity after 6 h and lost only 10 % of its activity in 10 % IL/buffer mixture. Upon the confirmation of the PKC-Cel stability, EFB was subjected to IL-pretreatment followed by hydrolysis in a single step without further removal of the IL. The findings revealed that choline acetate [Cho]OAc and choline butyrate [Cho]Bu were among the best ILs used in the study since 0.332 ± 0.05 g glucose/g and 0.565 ± 0.08 g total reducing sugar/g EFB were obtained after 24 h of enzymatic hydrolysis. Compared to the untreated EFB, the amount of reducing sugar obtained after enzymatic hydrolysis increased by three-fold in the case of [Cho]OAc and [Cho]Bu, two-fold with [EMIM]OAc and phosphate-based ILs whereas the lowest concentration was obtained in [TBPH]OAc. Pretreatment of EFB with [Cho]OAc and [Cho]Bu showed significant differences in the morphology of EFB samples when observed with SEM. Analysis of the lignin, hemicellulose and hemicellulose showed that the total lignin content from the raw EFB was reduced from 37.8 ± 0.6 to 25.81 ± 0.35 % (w/w) upon employment of [Cho]OAc in the compatible system. The PKC-Cel from *T. reesei* (RUTC30) exhibited promising characteristics that need to be investigated further towards a single-step process for bioethanol production.

## Introduction

Lignocellulosic biomass has been attractive as a renewable and sustainable resource for biofuel production. Combined with an enzymatic hydrolysis, a process where enzyme converts the biomass to fermentable sugars, it offers the high yields to products vital to economic success (Yang et al. 2011). Despite its potential, lignocellulosic biomass has been quietly well-known for its high recalcitrant to chemical and biological degradation. Cellulose as the main component of lignocellulosic materials is sheltered by lignin and hemicelluloses that wrap the molecule (Laureano-Perez et al. 2005), making it difficult for enzymatic hydrolysis process to take place.

There are several pretreatment methods prior to hydrolysis process which could be employed to achieve cellulase-accessible substances, such as milling (da Silva et al. 2010), hot water treatment (Sreenath et al. 1999), steam explosion, acid (Sindhu et al. 2014) and alkaline hydrolysis (Zainan et al. 2013), irradiation (Ninomiya et al. 2013) and microbial treatment (Rashid et al. 2009). However, various drawbacks were reported for those approaches such as high energy input, cost, slow reaction and excessive degradation of products (Moran-Mirabal 2013).

Ionic liquids have been introduced recently as an alternative method to perform the pretreatment of lignocellulosic biomass. These are organic salts in liquid form at below 100 °C and have been receiving considerable attention as substitutes for volatile organic solvents. They are non-flammable, non-volatile, and recyclable with remarkable properties of outstanding solvating potential, thermal stability, and tunable properties by suitable choices of cations and anions (Ghandi 2014). Ionic liquids serve as efficient tools for lignocellulose pretreatment in which they could disrupt the hydrogen bonds and expose the lignocellulosic material to the cellulase enzymes to facilitate the hydrolysis. A study by He et al. (2015) on the utilization of IL 1-butyl-3-methylimidazolium chloride ([Bmim]Cl) combined with HCl and water revealed a noticeable increase in reducing sugars after the successive stage of enzymatic saccharification. The yield of the reducing sugars observed after the pretreatment with IL was 95.1 % after a 48-h saccharification process, which is considered a great improvement compared to the untreated (25.6 %) and alkali-treated (82.2 %) corn stover.

Regardless of their potential, ILs come with a certain limitation when the enzymatic process is incorporated into the process (in situ). Many ILs induce different degrees of enzyme inactivation, and cellulose, as the enzyme with a function to hydrolyze cellulose, is not an exception. Ionic liquids may result in deactivation of cellulase, and this fact has been driving many types of research towards producing IL-compatible cellulases that can retain their activity in certain concentrations of the ILs. Some studies have reported commercial cellulase stability in several ILs as introduced by Wang et al. (2011) in 15 % [EMIM]OAc in the saccharification of yellow poplar biomass. Treatment of switchgrass was also reported in [EMIM]OAc followed by enzymatic hydrolysis at 10–20 % of the IL (Shi et al. 2013). Furthermore, cellulase enzyme stability was studied by the researchers to identify a compatible IL-enzyme system, such as in cholinium-based ILs (Ninomiya et al. 2015a). Another study of in situ application of cellulase was performed by He et al. (2016). The study showed that a compatible IL-cellulase system was developed with the employment of cellulase from *Galactomyces* sp. CCZU11-1 to media containing IL 1-methyl-3-methylimidazolium dimethylphosphate [Mmim][DMP]. The reducing sugar yield obtained at 62.1 % in the IL [Mmim]DMP—water media containing 20 % (w/v).

Aside from the enzymes’ compatibility, the high cost of ILs and the concern over the environment on the disposal of ILs are also some of the main obstacles hindering the large-scale application in the pretreatment of lignocellulosic biomass. Advantageously, ILs may have a good recyclability (Ninomiya et al. 2015c) and can be reused to reduce the cost and the impact on the environment. There are several methods to recycle ILs, and distillation of volatile solutes is always the first choice to recover the ILs due to their low vapor pressure. Other methods incorporated are extraction with organic solvents or supercritical carbon dioxide, as well as membrane separation processes. One could employ a method, but the recovered activity and the structure of the ILs might differ based on the type of the ILs used, the biomass type, and the pretreatment process engaged.

Considering the cost associated with commercial enzymes and ionic liquids, this study focused on obtaining locally produced cellulase by utilizing an abundant raw material, palm kernel cake (PKC), to serve as the media for fungal growth. Furthermore, turning to the synthesis of the ILs from their raw materials would cut the cost and increase the efficiency considering the possibility of recyclability of the ILs for several times. This approach could benefit the fermentable sugars production from lignocellulosic biomass for further applications such as bioethanol production at a lower cost in which the drawback of going through a multi-step process could be avoided. The cellulase enzyme (PKC-Cel) was tested for its compatibility with several ILs to seek its stability to be used without the requirement of regeneration. Although the report is not the first of its kind, however, these findings provide a contribution to the production of low-cost cellulase enzyme which is compatible with ILs, where this compatible IL-cellulase system is promising for the efficient pretreatment and subsequent hydrolysis of native biomass to produce biofuels in a single stage process.

## Methods

### Materials and strain collection

Palm kernel cake (PKC) and Empty fruit bunches (EFB) were collected from Sime Darby’s West Mill Plantation in Carey Island, Malaysia. Samples were washed, dried at 60 °C until constant weight obtained, then ground to 1.0 mm size. *Trichoderma reesei* (RUTC30) was purchased from American Type Culture Collection (ATCC) and revived as instructed. Other chemicals and solvents were obtained from Fisher Scientific, MERCK, and Bumi Pharma Sdn. Bhd, Malaysia. Commercial ionic liquids were purchased from MERCK and Sigma-Aldrich.

### Ionic liquids synthesis

Choline acetate [Cho]OAc and choline butyrate [Cho]Bu were prepared as described by Ninomiya et al. (2015a) in which an equimolar amount of acetic acid or butyric acid (Friedemann Schmidt Chemical, Sigma-Aldrich) was added dropwise to a choline hydroxide solution 45.0 wt% in methanol (Sigma-Aldrich) with cooling in an ice- bath. The mixture was stirred at room temperature for 6–12 h to complete the reaction. Water and methanol were removed under vacuum using a rotary evaporator (Buchi R215) at 40 °C (1 h, 337 mbar) and 90 °C (2 h, 314 mbar). The resultant residue was dried under vacuum overnight (Ninomiya et al. 2015a). Tertabutylphosphonium acetate [TBPH]OAc was prepared by adding an equimolar amount of acetic acid to tetrabutylphosphonium hydroxide solution (40 % in water) at room temperature. The reaction was continued for at least 12 h, and water was removed under vacuum. 1-ethyl-3-methylimidazolium hydrogen sulfate [EMIM]HSO_4_ was prepared as reported by Tajik et al. (2009) with a dropwise addition of one equivalent of concentrated sulphuric acid (97 %) to a cooled solution of 1-ethyl-3-methylimidazolium chloride (1 equivalent) in anhydrous dichloromethane. The mixture was refluxed for 48 h and the HCl by-product formed in the reaction was collected by dissolving it in deionized water at 0 °C (titration measured the acidity of the aqueous solution of NaOH as a control for completion of the reaction). Upon completion of the reaction, the solution was cooled to room temperature and the dichloromethane was removed using the rotary evaporator. The ionic liquids were dried in Freeze dryer (LABCONCO). 1-Ethyl-3-methylimidazolium diethylphosphate [EMIM]DEP, 1,3-dimethylimidazolium dimethylphosphate [DMIM]DMP and 1-Ethyl-3-methylimidazolium acetate [EMIM]OAc were purchased from MERCK, Germany.

### Enzyme production

Cellulase was produced locally by the utilization of PKC as the basal medium. Water was added to the dry PKC to achieve 65 % moisture content, and 2 % (w/w) of the spore suspension was added after sterilization. The fermentation was carried on at room temperature (30.0 ± 2 °C) and 7 days of solid-state fermentation (SSF). The crude enzyme was extracted with citrate buffer (pH 4.8 ± 0.2) followed by centrifugation. The enzyme was purified in a multi-step technique using cross-flow filtration. The free cell supernatant collected from the centrifugation was consigned to micro and ultra-filtration using hollow fiber membrane cartridge. A membrane of 0.45 μm with an active surface area of 0.011 m^2^ was utilized for the microfiltration procedure. Ultra-filtration was employed using ultra-filtration membranes (PALL, MWCO 30 and 10 Kd). The retentate containing the enzyme was collected and tested for enzyme activity. The activity in filter paper units (FPU) was monitored using Whatman no. 1 filter paper. One unit of FP was determined as 1 μmol of glucose liberated per ml enzyme per minute (Ghose 1987). Glucose liberated was calculated from the glucose standard curve. Endo-β-1,4-D-glucanase was measured by using carboxymethyl sodium salt (CMC) as a substrate (Salvador et al. 2010). Reducing sugar released by cellulase enzyme was calculated from the glucose standard curve. Protein content was determined by folin-phenol reagent method (Lowry et al. 1951) and calculated using bovine serum albumin (BSA) standard curve.

### Determination of PKC-Cel molecular weight by SDS-PAGE

The crude and the concentrated enzyme were both run on Bio-Rd Mini-Protean II Dual Slab Cell, following the given instructions. Sodium dodecyl sulphate polyacrylamide gel electrophoresis (SDS-PAGE) was performed on a 5 % stacking and a 12 % separating gel according to the method of Laemmli (1970) against the low range protein markers (BIO-RAD). After the electrophoresis, the gels were stained with Coomassie brilliant blue R-250 for visualization of protein bands.

### Effect of pH on PKC-Cel activity

The optimal pH for enzyme activity was determined by incubating the enzyme-substrate solution at different buffers: 50 mM sodium citrate buffer (pH 3.0, 4.0 and 5.0), phosphate-citrate buffer (pH 6.0), phosphate buffer (pH 7.0 and 8.0) and Tris–HCl buffer (9.0) using CMC as the substrate. Residual activity was determined by DNS method and expressed in percentage taking the control as 100 %. Control was incubated in 50 mM citrate buffer, pH 4.8.

### Effect of temperature on PKC-Cel activity and stability

The optimal temperature for PKC-Cel activity was determined by incubating the enzyme-substrate mixture at different temperatures ranging from 30° to 70 °C at the optimal pH from the previous step. For temperature effects on the enzyme stability, the PKC-Cel was pre-incubated from 30° to 70 °C, for 24 h. Residual activity was determined in percentage by recording the initial activity as 100 %.

### Stability of PKC-Cel in ionic liquids (ILs)

The synthesized and commercial ILs were tested for their compatibility with the PKC-Cel for a particular period. PKC-Cel was incubated in various ILs concentrations: 10, 20, 40, 60, 80 and 100 % (v/v). For the control, cellulase enzyme was incubated in citrate buffer (50 mM, pH 4.8 ± 0.2). Six ILs were tested; choline acetate [Cho]OAc, choline butyrate [Cho]Bu, 1-ethyl-3-methylimidazolium acetate [EMIM]OAc, 1-ethyl-3-methylimidazolium diethylphosphate [EMIM]DEP, 1,3-dimethylimidazolium dimethylphosphate [DMIM]DMP, tetrabutylphosphonium acetate and [TBPH]OAc. The reaction was conducted at the optimum temperature of PKC-Cel, 45.0 ± 2.0 °C. Samples were withdrawn every 1 h for 6 h and the activity was expressed as a residual activity using the enzyme/buffer solution as a control (100 %). The activity was assessed by DNS method as described in the enzyme production section.

### Development of IL-PKC-Cel system for biomass saccharification: pretreatment and saccharification of EFB

The EFB biomass (150 mg) was taken into a glass vial, and the IL was added to form a paste. The mixture was incubated at 90 °C for 60 min. The mixture was left to cool down to room temperature before buffer solution was added to achieve 10 % IL concentration, followed by the addition of the UF enzyme (100 U/g volatile solids). Hydrolysis stage was carried out for 24 h and reducing sugars were determined using DNS method. Glucose concentration was tested using glucose assay kit.

### Determination of structural carbohydrates and sugars

The cellulose, hemicellulose and lignin were determined as follows: 0.1 g of the sample was mixed with 2 mL of a 72 % (v/v) H_2_SO_4_ aqueous solution for 2 h at room temperature, then diluted with 75 mL of water, and autoclaved at 121 °C for 15 min. The acid-diluted hydrolysate was filtered, after which the amount of acid-insoluble lignin was determined by measuring the residue on the filter after drying at 100 °C for 12 h. The amount of acid-soluble lignin was determined from the ultra-violet (UV) absorbance of the filtrate at 205 nm (absorption coefficient of 110 L g^−1^ cm^−1^). The sum of the acid-insoluble and acid-soluble lignin was expressed as the total lignin amount. The amount of cellulose in raw and treated samples was determined by anthrone reagent colorimetric method (Updegraff 1969) using microcrystalline cellulose to prepare the standard curve. Upon IL treatment and hydrolysis, samples were centrifuged at 8000 rpm for 15 min, and the supernatant was tested for glucose and reducing sugar concentrations according to a glucose oxidase–peroxidase assay (Sadasivam and Manickam 1996) and the DNS method (Miller 1959) and expressed in gram per litre (g/L).The amount of hemicellulose was calculated from the xylose content multiplied by the correction factor of 132/150 (Ninomiya et al. 2015b).

The sample with the highest sugar yield (due to the analysis limitation) was tested for primary monosaccharides (glucose, xylose, and arabinose) in the hydrolyzate obtained from the enzymatic saccharification using HPLC on an Agilent 1200 HPLC system (California, USA) with a refractive index detector (RID) equipped with a REZEX RPM column (Phenomenex, USA) with water as the mobile phase and a fixed flow rate of 0.6 mL min^−1^, with isocratic elution and 60 °C (Abdul et al. 2016).

### Scanning electron microscopy (SEM) for raw and treated samples

All treated samples were dried using freeze dryer (LABCONCO) and stored at −80 °C. The dried samples were used for observation for scanning electronic microscope (SEM) by Hitachi SU1510 SEM at, 500X, 1000X and 5000X magnification at BSE mode.

### Fourier transform infrared spectroscopy (FTIR)

The ILs, IL-treated EFB and the hydrolysate were analyzed by FTIR within the range of 4000–500 cm^−1^ with 16 scans at a resolution of 4 cm^−1^. Spectral output was recorded in the absorbance mode as a function of wave number.

### IL recycling

Ionic liquids are considered as expensive solvents. Therefore, recycling of the IL from the mixture after hydrolysis is a critical step in the development of a cost-effective process. At the first trial in this study, the obtained mixture after hydrolysis was washed with acetone to precipitate the lignin, filtered through a 0.45 μm glass microfiber filter paper; then the acetone was evaporated followed by the aqueous layer under pressure using a rotary evaporator (Buchi R215). The aqueous solution was tested for sugar concentration using DNS method, and the weight of the recovered IL was recorded and compared with the initial one used for the pretreatment.

## Results and discussion

### Enzyme production

#### PKC-Cel productivity and activity

The production of cellulose was made by comparing three different substrates of palm oil kernel cake (PKC), rice husk (RH), and palm oil empty fruit bunches (EFB) for the culture growth of *Trichoderma reesei*. All substrates showed the competency on cellulase enzyme production with different levels of the activity (data not shown), and PKC was chosen for the further enzyme production.

**Fig. 1:**
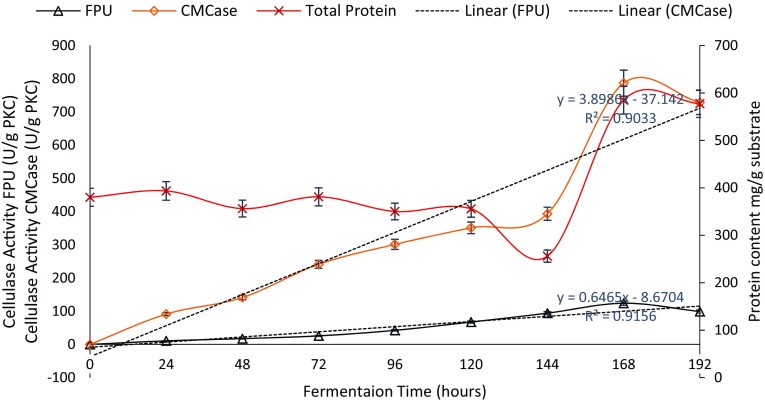
Determination of the time course for the fermentation of PKC by *Trichoderma reesei* and its effect on the activity and protein content of the extracted PKC-Cellulase (PKC-Cel)

In the current study, the activity and protein content were monitored for 8 days during the fermentation process. As illustrated in Fig. 1, the activity of PKC-Cel increased gradually with time to reach its maximum on the 7th day. Enzyme activity was calculated using both CMC and filter paper assays and resulted in 24.14 ± 1.82 (filter paper unit per milliliter) FPU/ml [123.33 ± 9.12 Unit (U)/gram dry substrate (gds)] and 157.872 ± 1.56 CMC unit/ml (789.386 ± 7.8 U/gds) after 7 days of fermentation.

The productivity per hour was determined from the slope after 7 days at 3.89 U g^−1^ h for CMCase and 0.65 U g^−1^ h for FPase. After 8 days of the fermentation process, the activity dropped noticeably to 145.67 ± 7.28 CMCase U/ml (728.325 ± 11.65 U/gds) and 19.806 ± 2.97 FPU/ml (99.033 ± 9.34 U/gds). The protein content of the crude enzyme was recorded during the fermentation process and reached its maximum (584.722 ± 21.28 mg/g PKC) on the 7th day and remained almost stable afterward. The protein concentration trend for the extract showed that the enzyme production increased simultaneously throughout the process. It can be suggested that consumption of available nitrogen sources is conducted during the process while some proteins are liberated into the medium in the form of the PKC-Cel enzyme and other proteolytic activities. The fluctuation in the protein concentration might be due to the presence of the soluble protein in the raw PKC. These changes may be related to the protein uptake by the fungal growth during the fermentation. The increment in the protein content afterward might be a result of the bioconversion of the sugars into mycelia protein. The slight reduction observed could be attributed to the protein degradation by some proteases in the fermentation media in which amino acids are released (Iyayi 2004).

#### Determination of molecular weight by SDS-PAGE

**Fig. 2:**
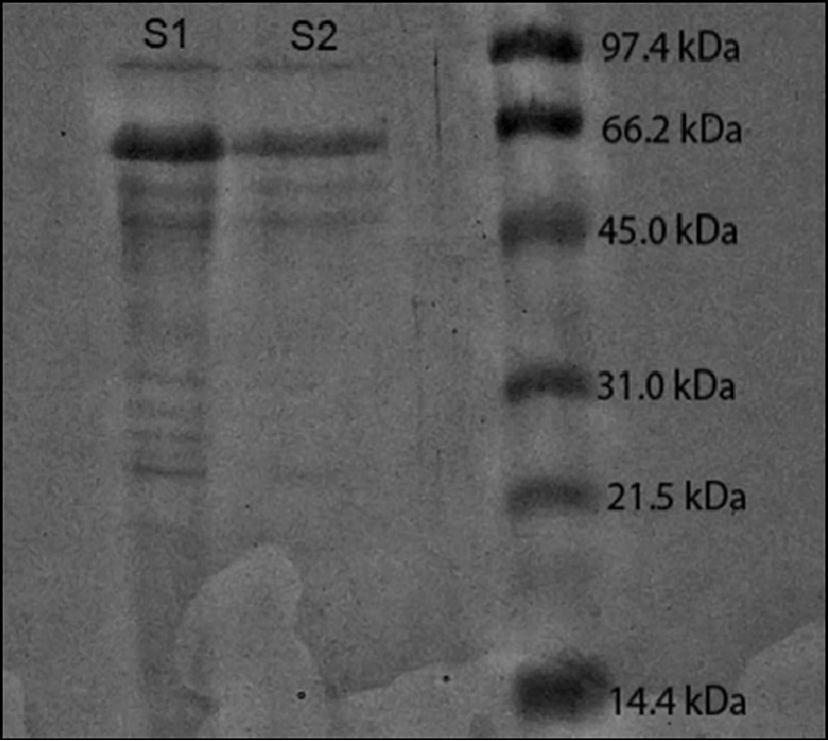
SDS-PAGE of: *Lane 1:* crude cellulase (S1), *Lane 2:* partially purified cellulase (S2) and *Lane 3:* molecular weight markers

Samples of both crude and partially purified PKC-Cel ran along against the low range protein markers. The purified PKC-Cel has a molecular weight range from 40 to 65 kDa as shown in Fig. 2. It is predicted that the enzyme is a mixture of carboxymethylcellulase (CMCases) or endo-β-glucanase, exo-β-glucanase and β-glucosidase. Remarking a comparison study, *Penicillium pinophilum* isolated from wood produced endoglucanase that has a molecular weight of 42 kDa after being purified from the extracted broth (Pol et al. 2012).

#### Effect of pH and temperature on PKC-Cel activity and stability

**Fig. 3:**
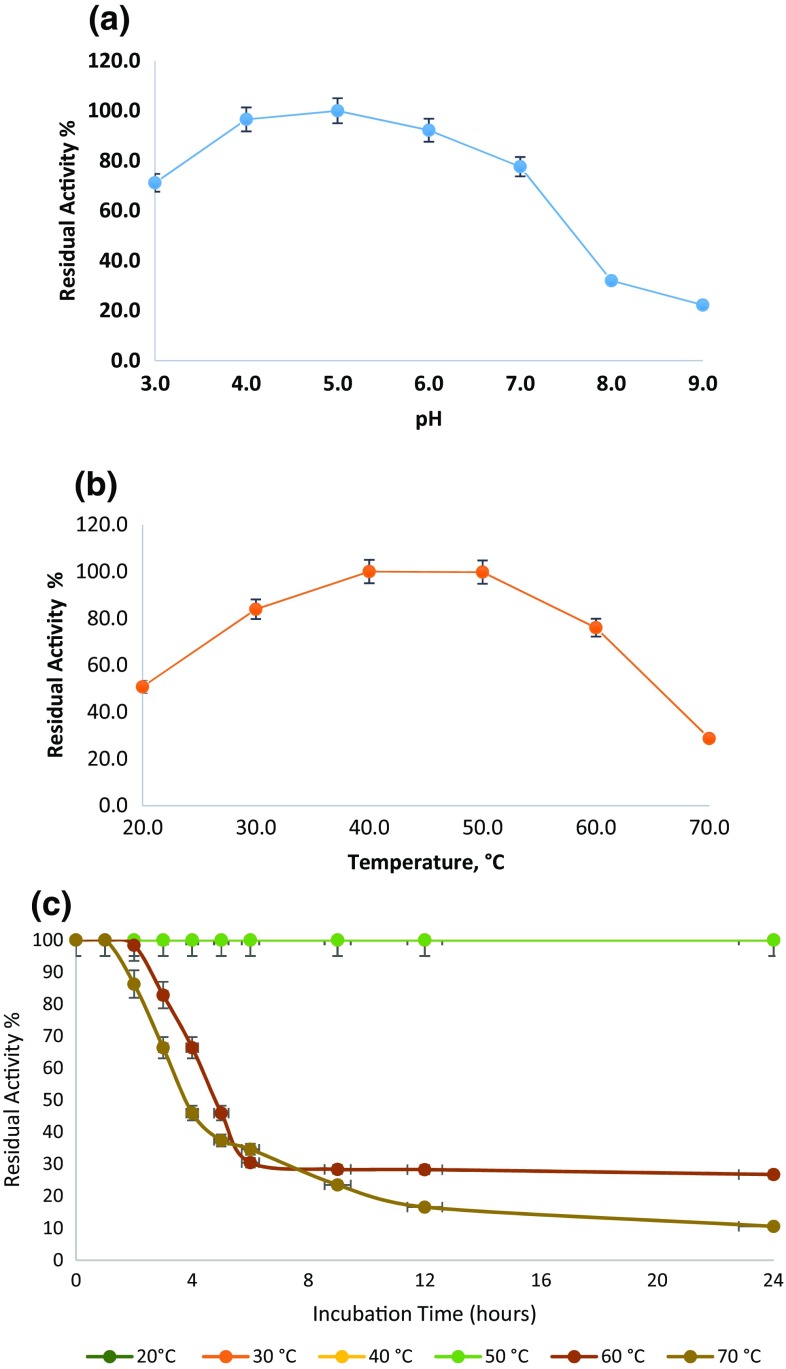
**a** Effect of pH on cellulase activity. **b** Effect of temperature on cellulase activity. **c** Effect of temperature on PKC-Cel stability for 24 h incubation period

PKC-Cel was active at all tested buffers where it exhibited its optimum at pH 5.0 using CMC as a substrate and at an optimum temperature of 45 °C (Fig. 3a, b). Many of the microbial and fungal cellulolytic enzymes have optimal activity in the pH 4–6 range and temperatures around 50 °C. A cellobiohydrolase from *N. koshunensis* worked optimally at pH 5.0 and 45 °C (Ni and Tokuda 2013).

In the observation, the PKC-Cel was exposed to different temperatures for different time intervals for 24 h. The enzyme was stable for 24 h at 20, 30, 40 and 50 °C maintaining its initial activity. However, at 60 °C, the PKC-Cel kept an activity of 82 % for 3 h and reduced to 26.73 % after 24 h. Likewise, higher temperatures (70 °C) resulted in activity reduction after two h to reach 82.2 and 10.53 % after 24 h as shown in Fig. 3c . The residual activity was analyzed by CMC method. It has been reported that cellulases originating from *Bacillus* strains were stable at 0–50 °C while at a higher temperatures (>70 °C) the enzyme activity declined to <50 % (Lin et al. 2012). The current results are in agreement with the  reported data.

### Development of IL-PKC cel system

#### Stability of PKC-Cel in ionic liquids

**Fig. 4:**
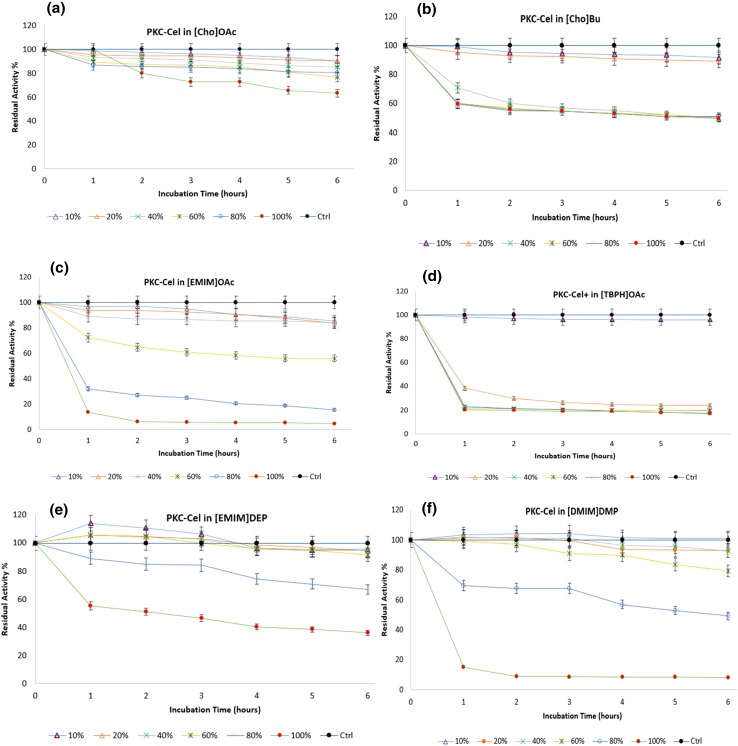
Compatibility of PKC-Cel with six different ionic liquids (ILs) for a period of 6 h at enzyme optimum conditions (pH 5.0 and 45 °C): **a** [Cho]OAc, **b** [Cho]Bu, **c** [EMIM]OAc, **d** [TBPHA]OAc, **e** [EMIM]DEP and **f** [DMIM]DMP

It is well known for ILs to dissolve cellulose and serve as reaction media for biocatalysis (Swatloski et al. 2002), yet it is also observed that residual IL in the recovered cellulose can affect the enzymatic hydrolysis by causing a loss of the activity due to the protein unfolding (Bose et al. 2010; Turner et al. 2003). Kamiya et al. (2008) used a series of dialkylphosphate ILs for the pretreatment of biomass as they showed less denaturing to proteins. Nevertheless, we studied the effects of few different ILs on the locally produced PKC-Cel in several concentrations as well as different time intervals (Fig. 4).

Six ILs were tested for their impact on the PKC-Cel; choline acetate [Cho]OAc, choline butyrate [Cho]Bu, 1-ethyl-3-methylimidazolium acetate [EMIM]OAc, 1-ethyl-3-methylimidazolium diethylphosphate [EMIM]DEP, 1,3-dimethylimidazolium dimethylphosphate [DMIM]DMP, tetra butyl phosphonium acetate [TBPH]OAc and 1-ethyl-3-methylimidazolium hydrogen sulphate [EMIM]HSO_4_.

In [Cho]OAc, the locally produced PKC-Cel retained more than 90 % of its activity at 10 and 20 % (IL/Buffer) for 6 h, 85 % and 80 % of the activity was maintained at 40 % (IL/Buffer) and at both 60 and 80 % (IL/Buffer), respectively. In 100 % (IL), 63.15 % of the activity remained after 6 h. In contrast, [Cho]Bu has shown stability up to 50 % of initial activity after 6 h in both 80 and 100 % of the IL. In lower concentrations, 10 and 20 %, the PKC-Cel maintained its activity at above 80 %. While in [EMIM]OAc, 85 % of the activity was preserved in concentrations between 10 and 40 % of the IL-aqueous solution for 6 h. At 60 % (IL/Buffer), 67 % of the activity was observed. However, higher concentrations result in a dramatic reduction in the activity since it reached 15 and 4.7 % at 80 % and the pure IL, respectively. Surprisingly, phosphate-based ILs showed different trends in which at the first 2 h, [EMIM]DEP activated the enzyme at concentrations ranging from 10 to 60 % (IL/Buffer) and more than 90 % of the activity was regulated for the next 6 h. PKC-Cel maintained 70 and 36 % of its activity at 80 and 100 % of the IL solution, respectively. Likewise, a similar trend was noticed for [DMIM]DMP (10–40 %) at the first 2 h while the activity reduction was observed in 60 % (IL-aqueous solution) as 80 % of the residual activity was recorded. In [TBPH]OAc, PKC-Cel maintained its activity at around 90 % in low concentrations, wherein higher concentrations, 20 % or less of the initial activity was recorded. In the case of [EMIM]HSO_4_, no activity of PKC-Cel was detected.

In summary, after 6 h of the incubation period, the maximum activity of PKC-Cel was observed in [Cho]OAc (>63 %) even at 100 % concentration of the IL and in [Cho]Bu (50 %) followed by [EMIM]DEP (36 %). For lower concentrations, the observed trend can be arranged as follows: [DMIM]DMP > [EMIM]DEP > [Cho]OAc > [Cho]Bu > [TBPH]OAc.

The extracellular cellulase produced by marine bacterium *Pseudoalteromonas* sp. by Trivedi et al. (2013) was studied for its activity and stability in six different ionic liquid; this study includes 1-ethyl-3-methylimidazolium methanesulfonate [EMIM][CH_3_-SO_3_], 1-ethyl-3-methylimidazolium bromide [EMIM]Br, 1-ethyl-3-methylimidazolium acetate [EMIM]OAc, 1-butyl-1-methylpyrrolidinium trifluoromethane sulfonate [BMPL]OTF, 1-butyl-3-methylimidazolium trifluoromethanesulfonate [BMIM]OTF and 1-butyl-3-methylimidazolium chloride [BMIM]Cl. The enzymatic activity was more than 90 % for all the ILs when used at concentration 5 % (v/v). At 20 % (v/v) IL concentration, the enzyme activity was at its maximum in [EMIM]OAc (94.37 %) > [BMPL]OTF (80.2 %) > [BMIM]OTF (74.69 %) > [BMIM]Cl (73.2 %) > [EMIM]Br (67 %) > [EMIM][CH_3_-SO_3_] (59 %). Furthermore, the residual activity of the studied enzyme (PKC-Cel) at higher concentrations (up to 60 % v/v) of [EMIM]OAc was comparable to other reports where the cellulases retained 86 and 76 % of the activity in the presence of 5 and 10 % [EMIM]OAc respectively (Wang et al. 2011), which proves that cellulases tend to lose some activities by increasing the IL concentration.

In most cases, it is also observed that ILs having hydrophobic nature, less viscosity, kosmotropic anion and chaotropic cation usually enhance the activity and stability of enzymes. However, the correlation could not be generalized because of many contradictory results (Naushad et al. 2012). [DMIM]DMP and [EMIM]OAc were both tested in enzymatic hydrolysis system and showed that ILs concentration above 40 % resulted in cellulase deactivation. At 90 % (v/v) of [DMIM]DMP, endoglucanase retained roughly 50 % of its activity (Wahlström et al. 2012). Similarly, in [EMIM]OAc, cellulase kept 40 % of the activity after 1 h and less than 1 % after 4 h (Ebner et al. 2014). Thus, in conclusion (1) smaller sizes of aromatic heterocyclic cation and smaller alkyl side chain boost up the cellulose dissolution. Overall, both (cation and anion) have an impact on cellulose solubility; hence, both components of the IL are equally important for cellulose dissolution (Badgujar and Bhanage 2015). Fukaya et al. (2008) denoted that the anionic component of ILs plays a fundamental role in enzymatic catalysis, and it was proved that ILs that contain alkyl-phosphate anions are capable of dissolving cellulose. Stabilization of cellulase in ILs is considered a promising approach for biomass treatment and saccharification of cellulose to a single-step continuous process. Therefore, the PKC-Cel might serve as an efficient biomass hydrolysis process hence; it is locally produced at a low cost by utilizing the agro-industrial wastes.

#### Biomass saccharification: pretreatment and saccharification of EFB

Different approaches have been adopted to convert lignocellulosic biomass into glucose before being used for bioethanol production, including physical, chemical, biological and physicochemical treatment (Balat 2011), and a pretreatment step is necessary to shorten the time required for the enzymatic conversion process. A preliminary study of 24 h-enzymatic hydrolysis was implemented to observe the potential IL to achieve the highest sugar yield as presented in Table 1. The study was subjected to a pretreatment in several ionic liquids which subsequently hydrolyzed without further washing.

**Table 1:** EFB pretreated and hydrolyzed samples analysis in different ILs-compatible system

Pretreatment	Acid soluble lignin % (w/w)	Acid insoluble lignin % (w/w)	Total lignin % (w/w)	Cellulose content % (w/w) after Pretreatment	Converted hemicellulose % (w/w)	Residual Hemicellulose content % (w/w)	Total reducing sugar after hydrolysis (g/L)	Glucose after hydrolysis (g/L)	Glucose yield g/g biomass	Total reducing sugar yield g/g biomass
Raw EFB-untreated	1.142 ± 0.1	36.80 ± 0.5	37.8 ± 0.6	2.08 ± 1.01	0.6 ± 0.005	23.062 ± 0.87	20.050 ± 0.1	0.123 ± 0.1	0.047 ± 0.01	0.115 ± 0.05
[Cho]OAc	0.91 ± 0.05	24.90 ± 0.3	25.81 ± 0.35	32.64 ± 1.5	21.384 ± 0.2	4.841 ± 1.67	63.377 ± 0.1	24.86 ± 0.5	0.332 ± 0.05	0.565 ± 0.08
[Cho]Bu	1.10 ± 0.05	29.01 ± 0.5	30.11 ± 0.55	26.24 ± 0.54	15.664 ± 0.2	5.291 ± 2.05	60.235 ± 0.1	22.70 ± 0.1	0.303 ± 0.06	0.481 ± 0.8
[EMIM]OAc	1.01 ± 0.05	30.05 ± 0.4	31.06 ± 0.45	9.851 ± 0.72	13.024 ± 0.5	11.581 ± 0.75	39.787 ± 0.2	18.38 ± 0.1	0.125 ± 0.01	0.273 ± 0.05
[TBPH]OAc	1.15 ± 0.05	33.90 ± 0.5	35.05 ± 0.55	10.551 ± 1.78	8.624 ± 0.3	10.134 ± 0.83	26.935 ± 0.3	8.216 ± 0.1	0.056 ± 0.05	0.154 ± 0.04
[EMIM]DEP	0.99 ± 0.05	32.67 ± 0.3	33.66 ± 0.35	12.530 ± 0.85	10.56 ± 0.2	12.832 ± 0.89	37.779 ± 0.2	14.06 ± 0.2	0.096 ± 0.04	0.216 ± 0.06
[DMIM]DMP	1.16 ± 0.05	28.35 ± 0.4	29.51 ± 0.45	11.881 ± 0.87	11.00 ± 0.2	11.998 ± 0.55	36.878 ± 0.5	14.70 ± 0.1	0.086 ± 0.01	0.211 ± 0.5

After pretreatment of EFB in the compatible ILs for 60 min, excluding [EMIM] HSO_4_ which deactivates PKC-Cel, the treated-IL was diluted with citrate buffer to the final concentration of 10 % IL (v/v). The cellulose was then added to the mixture. In the presence of 10 % IL at 24 h of the reaction, 60.24 and 63.38 g/L of the reducing sugar were produced in [Cho]Bu and [Cho]OAc respectively compared to 20.05 g/L of the untreated EFB, which formed only about 30 % of the ILs-hydrolysate product. The conversion difference between pre-treated and untreated EFB indicated that the IL-pretreatment disrupted the structure and improved accessibility of the enzymes to the substrate. The findings from this study clearly showed that [Cho]OAc and [Cho]Bu were compatible with the PKC-Cel produced in this work. This was proved by the reduction of the lignin concentration along the process.

It was demonstrated that ILs pretreatment enhanced the saccharification significantly. With [Cho]OAc pretreatment, 0.332 ± 0.05 g of glucose per 1 g of raw EFB was obtained. The yield was slightly lower in the case of [Cho]Bu (0.303 ± 0.06 g/g EFB). [EMIM]OAc gave a comparable yield as well which reached 0.273 ± 0.05 g/g EFB. On the other hand, it was observed that [DMIM]DMP and [EMIM]DEP glucose yield was not as high as cholinium ILs. However, the lowest yield was recorded for [TBPH]OAc.

The partial removal of lignin and hemicellulose enhanced the subsequent enzymatic saccharification. In general, the anions in ILs serve as hydrogen bond acceptors, which interact with the hydroxyl groups in cellulose to make the weaken the crystalline structure of cellulose, while the cations interact with lignin via hydrogen bonds as well as π–π interaction (Asakawa et al. 2015). As the highest sugar concentration of reducing sugar was observed in [Cho]OAc, samples were subjected to primary monosaccharides analysis as described in the methods and compared to the untreated hydrolysate from EFB. As obtained from the results, the sample treated with [Cho]OAc released 24.011, 22.457 and 0.0896 g/L of glucose, xylose and arabinose respectively. In contrast, the untreated EFB hydrolysate released only 0.12347, 0.50044 and 0.1284 g/L of glucose, xylose, and arabinose. As observed from the total reducing sugars, the total is not equal to the obtained results which directed to the conclusion that there should be other types of reducing sugar presented in the hydrolysate such as galactose, glyceraldehyde, fructose, and ribose. The reducing sugars determined in the DNS method might also be derived  from cellobiose or maltose.

It has been explained that with the pretreatment, anions from ILs and hydrogen atoms of the hydroxyl group in cellulose macromolecular chain form hydrogen bonding, in which a hydrogen bonding network of cellulose will be interrupted, and the cellulose chain becomes fully exposed, so the reaction rate becomes faster and reducing sugar yield increases. At the end of the reaction a part of cellulose does not undergo hydrolysis, but the crystallinity is significantly lowered, and the structure becomes loose, so hydrolysis in aqueous solution can improve the reducing sugar yield (Zhang et al. 2015). Moreover, IL pretreatment has the potential to enhance the enzymatic digestibility of cellulose three times higher compared to the non-IL treated lignocellulose substrates due to the increase in porosity of the substrate and solubilization and redistribution of lignin components. This enhancement in digestibility of cellulose is attributed to the decrease in the crystallinity of cellulose. During and after the IL-treatment, the structure of cellulose undergoes polymorphic transformations. The changes in the structure of cellulose affect the accessibility of the substrate to the cellulase enzyme. Pretreatment can also influence hemicellulose hydrolysis, lignin dissolution or redistribution and increase the porosity of constituent polysaccharides (cellulose and hemicellulose) providing access to hydrolytic enzymes. The crystalline structure of native cellulose fibers, called cellulose I can be a major impediment to its hydrolysis to monomeric sugars. Conversion of cellulose fibers to other crystal forms, such as cellulose II or amorphous forms can significantly enhance their susceptibility to hydrolysis (Samayam et al. 2011).

#### Biomass saccharification: changes on cellulose, hemicellulose, and lignin

Ionic liquids exhibit the capacity to disrupt the structure of cellulose, hemicelluloses, and lignin of the lignocellulosic biomass. The outcome may vary from one type of IL to another, and from the six ILs employed on this research, all contribute to the changes in the cellulose, hemicelluloses, and lignin contents. Moreover, the ILs pretreatment results may as well influence the hydrolysis process, altering the extent of the total reducing sugar and glucose as its products. All data of cellulose, hemicelluloses, and lignin content, and their hydrolyzed products from raw, pre-treated, and hydrolyzed EFB are available in Table 1.

The cellulose content of the biomass after the pretreatment varies based on the ILs type employed for the process. The available cellulose content of the raw EFB observed at 2.08 ± 1.01 % (w/w), and the amount increased as ILs used. Pretreatment process by [Cho]OAc provides the highest available cellulose content for the hydrolysis process of the amount of 32.64 ± 1.5 % (w/w), while [EMIM]OAc appeared to give the least at 9.851 ± 0.72 % (w/w).

In the absence of the ILs pretreatment, the conversion process of hemicellulose seems to have ceased, with the amount of converted hemicelluloses at 0.6 ± 0.005 % (w/w) for the raw EFB. However, ILs pretreatment appeared to be promoting the conversion process and increase the converted amount for each pretreatment condition. The highest conversion process resulted in the amount of converted hemicelluloses at 21.384 ± 0.2 % (w/w) by [Cho]OAc and the lowest amount at 8.624 ± 0.3 % (w/w) by [TBPH]OAc.

In the removal of lignin content to facilitate the hydrolysis stage, ILs pretreatment carries a very considerable and vital task. The removal of lignin ensures the easiness of the enzyme to take place during the hydrolysis process. The total lignin content from the raw EFB was observed at 37.8 ± 0.6 % (w/w). This amount was reduced with a variation according to the ILs pretreatment employed. The lowest total lignin content could be observed in the employment of [Cho]OAc at 25.81 ± 0.35 % (w/w). At the opposite, total lignin content could be observed at its highest at the application of [TBPH]OAc at 35.05 ± 0.55 % (w/w).

The ILs pretreatment stage affected the nature of the hydrolysis stage, by either providing a readily to be degraded substrates, or the stability of the enzymes for the conversion process. The results can be reflected through the total reducing sugar and glucose after the hydrolysis process.

Total reducing sugar for the raw-hydrolyzed EFB was 20.050 ± 0.1 g/L. This amount was increased by the employment of ILs in the pretreatment stage. The highest total reducing sugar observed was 63.377 ± 0.1 g/L at the employment of [Cho]OAc while the lowest conversion was found at 26.935 ± 0.3 g/L of total reducing sugar by the employment of [TBPH]OAc. In a similar pattern, the highest observable glucose content after hydrolysis was 24.86 ± 0.5 g/L by the application of [Cho]OAc, and the lowest at 8.216 ± 0.1 g/L by [TBPH]OAc application.

### Scanning electron microscopy (SEM) for raw and pre-treated samples

**Fig. 5:**
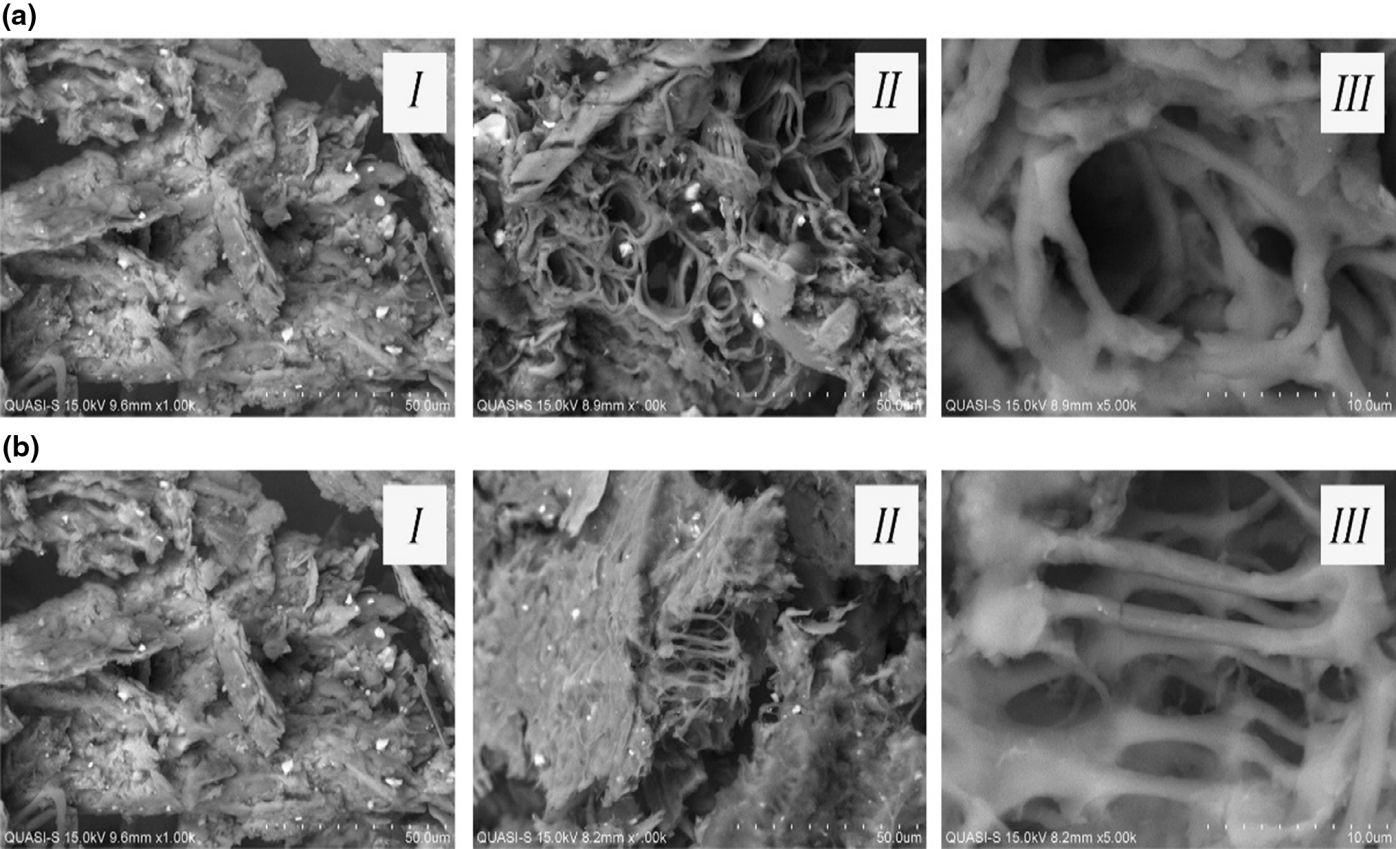
**a** Scanning electron micrographs of EFB samples in the (*I*) raw state (untreated) at ×1000; (*II*) [Cho]OAc pretreated at ×1000; and (*III*) [Cho]OAc pretreated at ×5000 magnification. **b** Scanning electron micrographs of EFB samples in the (*I*) raw state at ×1000; (*II*) [Cho]Bu pretreated at ×1000; and (*III*) [Cho]Bu pretreated at ×5000 magnification

Two ILs pre-treated samples were analyzed for their structure and morphology through scanning electron microscopy. Micrographs were generated for raw and ILs pre-treated samples in the utilization of [Cho]OAc and [Cho]Bu, which are previously shown as compatible with PKC-Cel. Both ILs provided a similar result in the resulting structures and characteristics of the pre-treated samples with a modest differentiation. In the application of [Cho]OAc, which is shown in Fig. 5a, the pre-treated samples showed deformed and corrugated structures on the surface compared to the raw samples which appeared to be rigid and sturdy in form. The succeeding step of pretreatment showed the residue of the cellulose fibers after an hour of exposure time to IL. Figure 5b shows a similar result on the EFB samples by the application of [Cho]Bu and PKC-Cel. The ILs pre-treated samples were corrugated on the surface structure, quite similar to the result of [Cho]OAc pre-treated samples. A slight dissimilarity appears only on the structure of the residue after pretreatment, where less visible cellulose fibers were captured. Both [Cho]OAc and [Cho]Bu showed significant differences in the morphology of EFB samples; before and after the treatment with the ILs. These were predicted as the result of the partial removal of lignin and the decreased cellulose crystallinity, thus revealing the disruption of tissue network.

### Fourier transform infrared spectroscopy (FTIR)

**Fig. 6:**
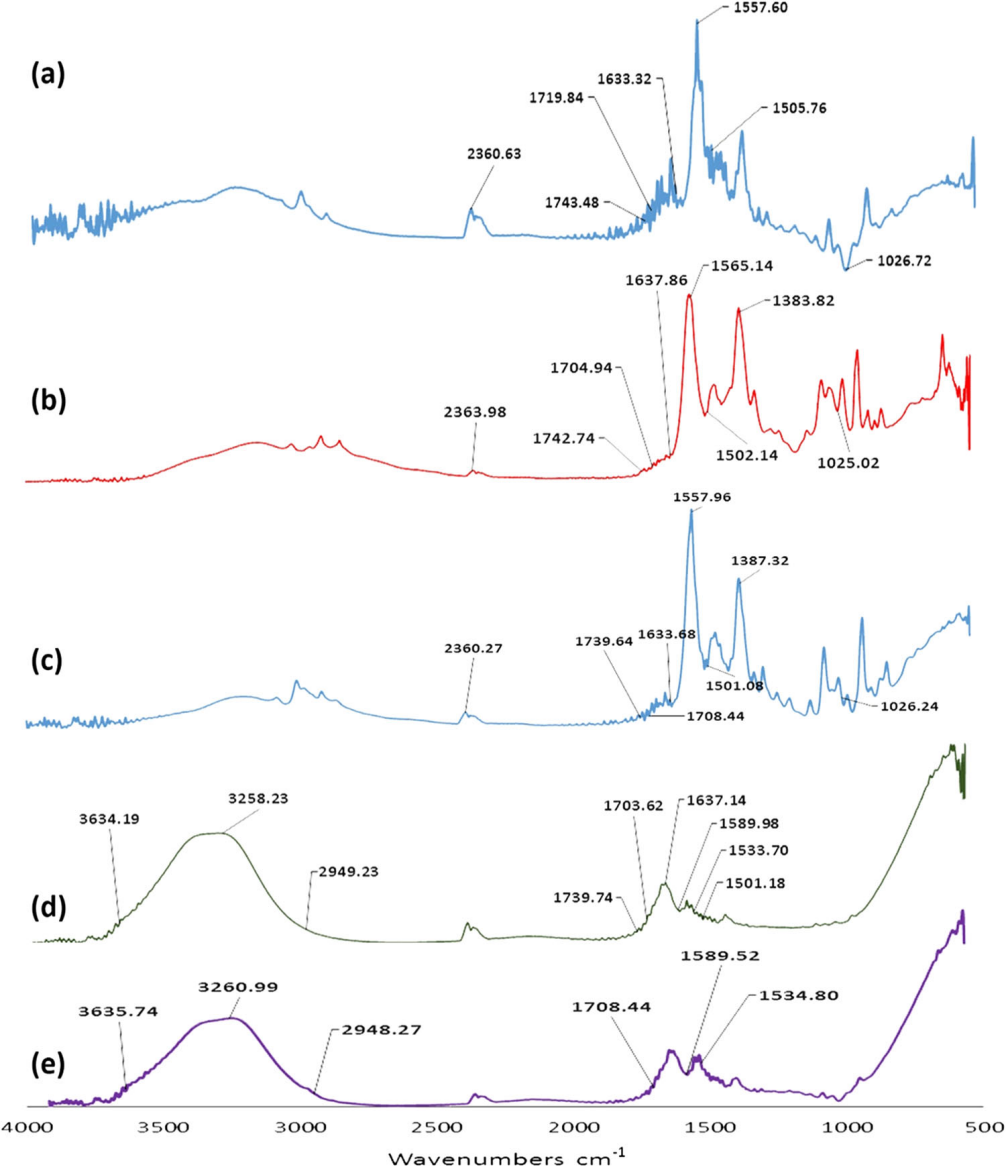
FTIR spectra of: **a** raw, **b** [Cho]OAc pretreated, **c** [Cho]Bu pretreated, **d** PKC-Cel hydrolysed EFB after upon [Cho]OAc pretreatment and **e** PKC-Cel hydrolysed EFB after upon [Cho]Bu pretreatment, at the absorbance bands of 500–4000 cm^−1^

Figure 6 shows the stacked FTIR spectra of raw, ILs pre-treated, and PKC-Celhydrolyzed samples by [Cho]OAc and [Cho]Bu pretreatment. In a comparative study conducted by (Abdul et al. 2016) with oil palm EFB treated with ammonia fiber expansion, similar patterns were exhibited where lignin and hemicellulose structures were predicted to be at 1800–900 cm^−1^. In this study, the peak absorbance bands showed a notably decreased intensity in the range of 1505–1557 cm^−1^ in the ILs pre-treated samples. A similar result from [Cho]Bu pre-treated samples also provides a similar intensity at absorbance bands of 1503–1556 cm^−1^, as illustrated in Fig. 6. The peak changes were predicted to be related to C=C stretching vibration of the aromatic ring of lignin. The pretreatment with [Cho]OAc and [Cho]Bu was partially providing an impact in the removal of lignin from all pre-treated materials.

Changes can also be observed at the absorbance bands of 1720–1740 cm^−1^ of [Cho]OAc pre-treated samples and the similar range of 1720–1739 cm^−1^ of [Cho]Bu applications in the comparison to the related bands of raw materials. The peak changes, with reduced peak intensities, were related to a C–O–C asymmetric bridge stretching vibration in hemicellulose, as well as a C–H stretching of cellulose. This is also supported by the result of Abdul et al. (2016) which mentioned that the peaks at 1738 cm^−1^ of EFB correlated to carboxylic and carbonyl bond which is commonly found in hemicellulose. A prominent peak of 1725–1730 cm^−1 ^was also observed in the spectra of oil palm frond biomass (OPFB) pre-treated with [EMIM]DEP, which was claimed to be attributed to polysaccharides. Despite the slight differences between the bands spectra, the study showed that [EMIM]DEP was efficient in the removal of lignin and hemicellulose content (Financie et al. 2016).

It was indicated that the pretreatment caused the dissolution of carbohydrates during the process of ILs pretreatment (Trinh et al. 2015). Further analysis by enzymatic hydrolysis is also provided for each type of ionic liquids. Similar results are observed for most types of ionic liquids, indicating the availability of cellulose materials to be converted by PKC-Cel. Samples spectra from the hydrolyzed biomass after [Cho]OAc and [Cho]Bu pretreatment showed the presence of reducing sugar through its distinctive bands in the range of 2947–3633 and 1707–1584 cm^−1^. The capability to produce reducing sugars provides the indication that all types of ionic liquids in this study are compatible with PKC-Cel. The possibility of retaining the stability of the locally-produced PKC-Cel shows a promising compatibility in a single-step hydrolysis process. However, the presence of other constituents and the concentration of reducing sugars from each type, ILs pre-treated samples leave a notable remark for each ILs-PKC-Cel stability.

### Ionic liquids recovery

**Fig. 7:**
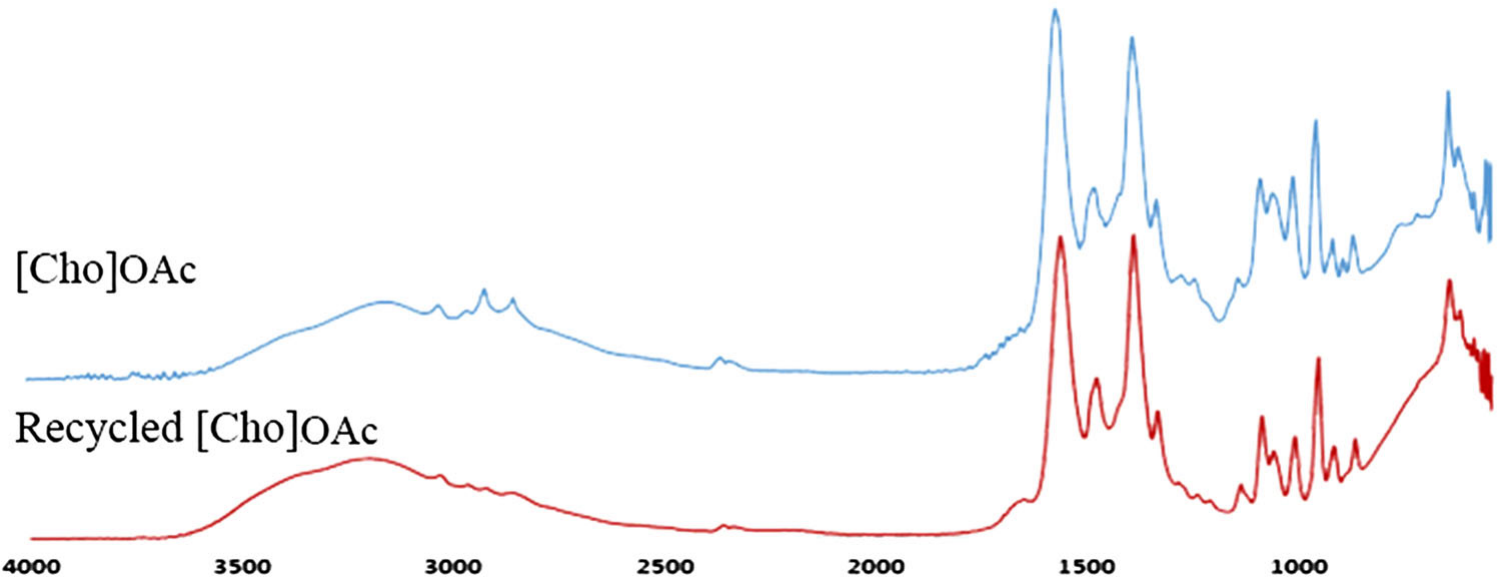
FTIR spectra of initial and recycled [Cho]OAc at the absorbance bands of 500–4000 cm^−1^

Ionic liquids recovery was performed only for [Cho]OAc, which produced the highest reducing sugar during the hydrolysis process. The preliminary results showed that over 75 % of [Cho]OAc was successfully recovered based on the weight obtained and appearance. As shown in Fig. 7, no structural changes in the FTIR analysis of both original and recovered IL after hydrolysis may be observed. At this point, it can be concluded that [Cho]OAc can be reused for biomass pretreatment for an efficient process to be executed.

Ionic liquids can be recovered from the hydrolysate using several techniques which include nanofiltration, electrodialysis, ion-exclusion chromatography and liquid–liquid extraction (Ninomiya et al. 2015b). Despite the potential, recovery and reuse of ILs are essential to make the process economically feasible. ILs are still more expensive than the conventional pretreatment solvents. The recycling of ILs up to 10–20 times was proposed to allow for cost per that is comparable to conventional solvents, thus making ILs cheaper alternatives as reusable solvents. ILs are comparatively easy to recycle by evaporation or distillation of the anti-solvents due to the low volatile nature of ILs. It has been reported that the ILs can be recovered and reused at least up to 5–7 times without reduction in their efficiency (Ninomiya et al. 2015d; Shi et al. 2013). However, recovery of ILs is still a matter of concern considering the scaled up production of ILs. Furthermore, ILs that have undergone pretreatment composed of dissolved IL and the anti-solvent, in addition to soluble biomass compounds (e.g., lignin, soluble carbohydrates with low molecular weight, degradation products, extractives and others) that were not precipitated. Recovery of these dissolved compounds is necessary; for example, the recovered lignin may serve as a potential raw material for the production of polymeric substances (Soudham et al. 2015).

## Conclusions

The PKC-Cel exhibited promising characteristics as it showed a high activity at pH 5.0 and 45 °C that need to be investigated further towards a one-step process for bioethanol production. From all ionic liquids tested in this study, [Cho]OAc and [Cho]Bu were found to be the ionic liquids which provide more stability in PKC-Cel-ILs system. Based on the findings, this system is promising and should be further developed and optimized for an efficient process to be achieved. It is recommended to establish a cost effective technique for the IL recovery from the sugar solution. Moreover, it will be more efficient to be able to reuse both the enzyme and the IL after hydrolysis in order for use to claim the cost effectiveness process. Further analysis needs to be performed to evaluate the cost compared to the conventional solvents. Compatibility of PKC-Cel with ionic liquids is also another issue to be addressed in the application of PKC-Cel-ILs in bioethanol production.

## Abbreviations

PKC-Cel: Palm kernel cake cellulase
[Cho]OAc: Choline acetate
EFB: Empty fruit bunch
[Cho]Bu: Choline butyrate
CMC: Carboxymethyl cellulose
[TBPH]OAc: Tertabutylphosphonium acetate
[EMIM]DEP: 1-Ethyl-3-methylimidazolium diethylphosphate
[DMIM]DMP: 1,3-Dimethylimidazolium dimethylphosphate
[DMIM]DMP: 1,3-Dimethylimidazolium dimethylphosphate
gds: Gram dry substrate
